# Double chamber-like left ventricle

**DOI:** 10.1093/ehjcr/ytae459

**Published:** 2024-08-27

**Authors:** Mikio Shiba, Yoshiharu Higuchi

**Affiliations:** Cardiovascular Division, Osaka Police Hospital, 10-31 Kitayama-cho, Tennoji-ku, Osaka 543-0035, Japan; Cardiovascular Division, Osaka Police Hospital, 10-31 Kitayama-cho, Tennoji-ku, Osaka 543-0035, Japan

**Figure:**
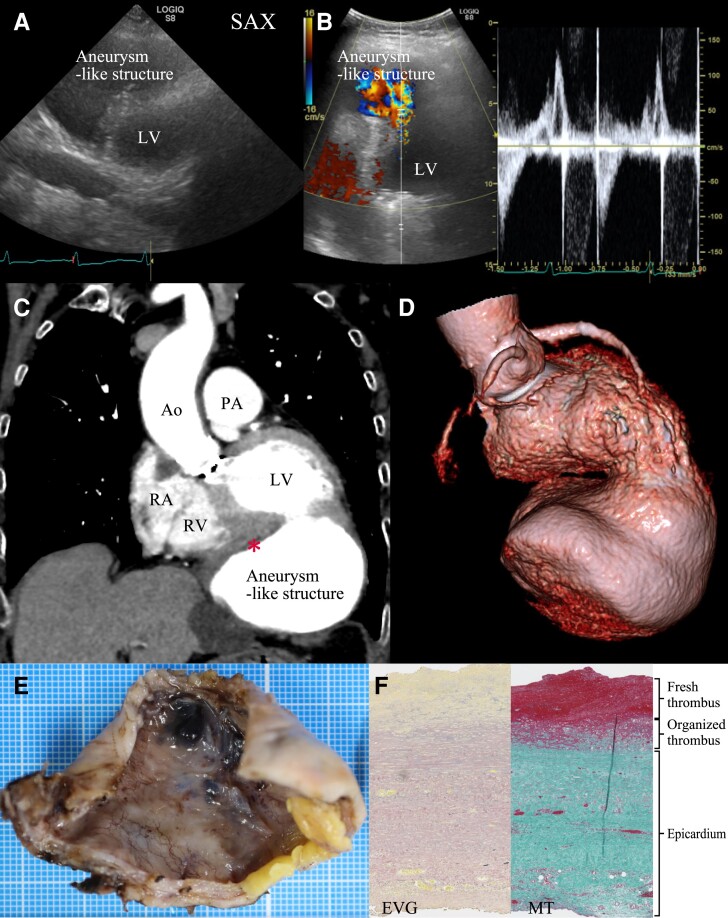


An 89-year-old woman who had undergone surgical aortic valve replacement 25 years ago was admitted to our hospital with deficient articulation and diagnosed with a cerebrovascular accident. An echocardiogram revealed what appeared to be a ventricular aneurysm (*Panel A* and see Supplementary material online, *Movie S1*), with a to-and-fro flow observed between it and the left ventricle (*Panel B* and see Supplementary material online, *Movie S2*). A contrast-enhanced thoracic computed tomography (CT) scan indicated a left ventricular aneurysm 90 mm in diameter, with a mural thrombus that was very thin and stratified (*Panels C* and *D*). On history taking, it was revealed that 4 months ago, she had received conservative treatment for a ventricular rupture secondary to acute myocardial infarction due to the occlusion of the right coronary artery. The aneurysmal structure was surgically resected (*Panel E*), and the heart was repaired with a bovine pericardial patch. Histological examination led to a diagnosis of a pseudoaneurysm (*Panel F*).

The patient was discharged without complications after a month of hospital stay. Pericardial adhesions resulting from her previous open-heart surgery may have provided structural support, preventing rupture and allowing the pseudoaneurysm to enlarge over time. Thrombus formation in a pseudoaneurysm is one of the causes of embolic stroke. In addition to imaging diagnostics, such as echocardiography and CT, a detailed medical history provides key insights into the mechanism of anatomical deformation and subsequent thrombus formation.

## Supplementary Material

ytae459_Supplementary_Data

## Data Availability

The data underlying this article will be shared upon reasonable request to the corresponding author.

